# Genome-wide association study for grain yield and component traits in bread wheat (*Triticum aestivum* L.)

**DOI:** 10.3389/fgene.2022.982589

**Published:** 2022-08-26

**Authors:** Hanif Khan, Gopalareddy Krishnappa, Satish Kumar, Chandra Nath Mishra, Hari Krishna, Narayana Bhat Devate, Nagenahalli Dharmegowda Rathan, Om Parkash, Sonu Singh Yadav, Puja Srivastava, Suma Biradar, Monu Kumar, Gyanendra Pratap Singh

**Affiliations:** ^1^ ICAR-Indian Institute of Wheat and Barley Research, Karnal, India; ^2^ ICAR-Sugarcane Breeding Institute, Coimbatore, India; ^3^ ICAR-Indian Agricultural Research Institute, New Delhi, India; ^4^ Punjab Agricultural University (PAU), Ludhiana, India; ^5^ University of Agricultural Sciences, Dharwad, India; ^6^ ICAR-Indian Agricultural Research Institute, Jharkhand, India

**Keywords:** wheat, GWAS, SNPs, candidate genes, mapping, yield component traits

## Abstract

Genomic regions governing days to heading (DH), grain filling duration (GFD), grain number per spike (GNPS), grain weight per spike (GWPS), plant height (PH), and grain yield (GY) were investigated in a set of 280 diverse bread wheat genotypes. The genome-wide association studies (GWAS) panel was genotyped using a 35K Axiom Array and phenotyped in five environments. The GWAS analysis showed a total of 27 Bonferroni-corrected marker-trait associations (MTAs) on 15 chromosomes representing all three wheat subgenomes. The GFD showed the highest MTAs (8), followed by GWPS (7), GY (4), GNPS (3), PH (3), and DH (2). Furthermore, 20 MTAs were identified with more than 10% phenotypic variation. A total of five stable MTAs (*AX-95024590, AX-94425015, AX-95210025 AX-94539354*, and *AX-94978133*) were identified in more than one environment and associated with the expression of DH, GFD, GNPS, and GY. Similarly, two novel pleiotropic genomic regions with associated MTAs i.e. *AX-94978133* (4D) and *AX-94539354* (6A) harboring co-localized QTLs governing two or more traits were also identified. *In silico* analysis revealed that the SNPs were located on important putative candidate genes such as *F-box-like domain superfamily, Lateral organ boundaries, LOB, Thioredoxin-like superfamily Glutathione S-transferase*, *RNA-binding domain superfamily*, *UDP-glycosyltransferase family, Serine/threonine-protein kinase*, *Expansin*, *Patatin, Exocyst complex component Exo70, DUF1618 domain, Protein kinase domain* involved in the regulation of grain size, grain number, growth and development, grain filling duration, and abiotic stress tolerance. The identified novel MTAs will be validated to estimate their effects in different genetic backgrounds for subsequent use in marker-assisted selection (MAS).

## Introduction

Bread wheat (*Triticum aestivum* L., 2n = 6x = 42) is one of the most important staple food and the world’s highest-grown and traded cereal. It provides about 21% of calories and 19% of day-to-day protein to approximately 4.5 billion global populations (Braun et al., 2010). The annual gain in wheat yield should be increased from the current level of around 1%–1.6% to meet the food demand of the estimated global population of 9 billion by the year 2050 (Wheat Initiative, 2013; FAO, 2017). The available resources likely be reduced to a great extent; the problem will be further complicated by the erratic rainfall, reduced water table, change in temperature, and reduced soil health. For sustainable crop production, we need to increase the yield potential, and multiple stress tolerance and improve input use efficiency along with climate-smart agronomic practices (Giraldo et al., 2019). Integration of modern plant breeding tools like marker-aided selection (MAS), marker-assisted recurrent selection (MARS), genomic selection, and speed breeding with conventional breeding approaches is of paramount importance to enhance yield gain in wheat (Krishnappa et al., 2021).

Grain yield is a genetically complex trait and is an outcome of the combined effect of several agro-morphological and physiological traits (Chen et al., 2012; Sukumaran et al., 2018). Agro-morphological traits include grain number per spike, thousand kernel weight, biomass, harvest index, productive tillers number, spike length, grain weight per spike, and plant height has a significant effect on wheat grain yield along with phenological traits like days to heading, maturity, and grain filling duration (Sun et al., 2017; Wang et al., 2017; Liu et al., 2018; Ma et al., 2018; Jamil et al., 2019; Li et al., 2020). Unlike grain yield, many of the yield component traits have high heritability and are easier to select particularly during the early stages of breeding cycles. The yield plateaus may be avoided by selecting the yield components, as they offer additional avenues for genetic gain enhancement. It is suggested for trait-based breeding using elite and genetically complementary genotypes to enhance wheat yield improvement (Liu et al., 2015; Reynolds et al., 2017). The grain yield and its component traits are complex and quantitative, as each of these traits is controlled by several genes with small effects. Furthermore, most of the traits have low to moderate heritability with significant genotype × environment interactions (Kaya and Akcura, 2014). Molecular breeding is a potential strategy to improve complex traits like yield and its contributing traits, but a better understanding of genetic architecture is important for the effective utilization of molecular tools. Therefore, genetic dissection of agro-morphological traits is essential for the improvement of wheat yield.

Two approaches i.e. genome-wide association studies (GWAS) and quantitative trait loci (QTL) mapping are widely used methods to dissect the genetic basis of complex quantitative traits in crop plants. In the past decade, extensive efforts have been made to identify QTLs associated with grain yield and its component traits (Gao et al., 2015; Zhang et al., 2016; Jin et al., 2020; Isham et al., 2021; Kang et al., 2021; Li et al., 2022) in wheat through bi-parental populations based QTL mapping. Conventional QTL mapping mainly depends on structured populations like recombinant inbred lines (RIL), back-crosses (BC), and doubled haploids (DH). The several shortcomings associated with QTL mapping are low resolution due to one or few cross-overs and low marker density (Korte and Farlow, 2013). Recent advances in sequencing technologies created valuable genomic resources including high-quality genome data (Brenchley et al., 2012; Chapman et al., 2015), as result, several high throughput SNP arrays have been developed and utilized in wheat. GWAS becomes a complementary strategy to QTL mapping to dissect complex traits, particularly after the availability of large-scale genomic resources (Saidou et al., 2014). Unlike bi-parental population-based QTL mapping, GWAS consists of more genetically diverse lines that harbor several historical and ancestral recombination events. Additionally, the use of diverse germplasms as study materials has the potential to capture superior alleles that have been missed by breeding practices. GWAS is based on the linkage disequilibrium (LD) that has formed in a population over the generations, the genomic regions harboring QTLs can be detected even in the absence of inclusion of causal mutations among the set of available molecular markers.

The two common limitations (i.e. limited allelic diversity and low genomic resolution) associated with the bi-parental QTL mapping methods can be overcome in the GWAS approach (Brachi et al., 2011). However, the major challenge for GWAS is the control of false positives caused by population structure and family relatedness (Kaler et al., 2020). Incorporation of these two confounding factors as covariates in the mixed linear model (MLM) addressed the issue of false positives (Price et al., 2006), however, false negatives have been significantly increased which might exclude the important loci. To overcome the false negatives, multi-locus GWAS methods like multi-loci mixed linear model (MLMM), fixed and random model circulating probability unification (FarmCPU), and Bayesian-information and linkage-disequilibrium iteratively nested keyway (BLINK) have been developed (Zhang et al., 2019). The statistical power of BLINK is superior and gives reduced false-positive discovery compared to many available GWAS models including SUPER and FarmCPU, as BLINK removes the assumption of equal distribution of causal genes in the whole genome (Huang et al., 2019).

GWAS has been successfully used in wheat to dissect the genetic basis of yield and its component traits. In previous studies, GWAS panels have been phenotyped in a range of production conditions including drought, irrigated, and salt stress to identify QTLs. Several drought-tolerance QTLs associated with grain yield and its component traits have been identified (Edae et al., 2014; Ain et al., 2015; Gahlaut et al., 2019; Suliman et al., 2021; Bennani et al., 2022; Said et al., 2022). Similarly, genomic regions governing yield and its attributing traits in normal irrigated production conditions were also identified (Sukumaran et al., 2015; Sun et al., 2017; Godoy et al., 2018; Liu et al., 2018; Ma et al., 2018; Sukumaran et al., 2018; Bajgain et al., 2019; Jamil et al., 2019; Li et al., 2019; Rahimi et al., 2019; Sheoran et al., 2019; Ward et al., 2019; Ali et al., 2020; Alqudah et al., 2020; Pang et al., 2020; Alemu et al., 2021; Eltaher et al., 2021; Gao et al., 2021; Malik et al., 2021; Saini et al., 2022; Zhang et al., 2022). Also, QTLs were identified in hostile soils under salt stress conditions for yield and related traits (Hu et al., 2021). Similarly, MTAs were also identified for biotic stresses (Vikas et al., 2022) and quality traits (Sandhu et al., 2021; Rathan et al., 2022) in wheat. Although several marker-trait associations (MTAs) were identified in different GWAS studies for yield and its component traits, there might be several false positives in most of the studies due to a very low threshold (−log_10_ *p*-value ≥ 3.0) fixation to consider the MTA as a significant. In only a few GWAS (Gahlaut et al., 2019; Eltaher et al., 2021; Malik et al., 2021; Zhang et al., 2022), the threshold–log_10_ *p* values were adjusted by the calculation of the corresponding Bonferroni correction at a significance level of 5% to reduce the false positives. In wheat, many QTLs/MTAs have been identified; however, additional genetic studies are warranted using different genetic materials, as we have not reached a saturation point (Singh et al., 2021). Thus, more efforts are required to dissect the genetic mechanisms of yield and component traits in wheat and to devise marker-based breeding approaches that involve marker-assisted selection or genome-wide selection to obtain increased genetic gains. The present study aimed to identify the genomic region (s) associated with grain yield and component traits i.e. days to heading (DH), grain filling duration (GFD), grain number per spike (GNPS), grain weight per spike (GWPS), grain yield (GY), and plant height (PH) in a panel of diverse bread wheat genotypes in a range of environments through the GWAS approach and the putative candidate genes associated with the SNPs.

## Materials and methods

### Plant material and field experiments

A set of 280 diverse bread wheat genotypes (Supplementary Table S1) consisting of advanced breeding lines and commercial cultivars were used for GWAS analysis. The GWAS panel of 280 genotypes was selected from the All India Coordinated Research Project on Wheat and Barley. The GWAS panel was evaluated at five different environments: E1- University of Agricultural Sciences, research farm, Dharwad (15°29′20″N, 74°59′3″E, 750 m AMSL), E2- ICAR-Indian Agricultural Research Institute, New Delhi (28°38′30″N, 77°09′58″E, 228 m AMSL), E3- ICAR-Indian Agricultural Research Institute, Jharkhand (24°16′58.4″N, 85°21′16.1″E, 651 m AMSL), E4- ICAR-Indian Institute of Wheat and Barley, Karnal (29°41′8″N, 76°59′25″E, 250 m AMSL), and E5- Punjab Agricultural University, Ludhiana (30^o^54’ N, 75^o^48′E, 247 m AMSL). The crop was sown in the first fortnight of November during the 2020–21 *Rabi* (winter) season under irrigated conditions. The genotypes were planted in an augmented block design with only the checks (DBW187, MACS6222, WH1124, and WH1142) repeated in a 2 row of 2 m length with a row spacing of 20 cm.

### Phenotyping and phenotypic data analysis

All the genotypes of a GWAS panel were phenotyped for six quantitative traits i.e. GWPS (gm), GNPS (number), GY (gm), DH (days), GFD (days); PH (cm) at Dharwad (E1), IARI-Delhi (E2) (except GNPS), IARI Jharkhand (E3), Karnal (E4) (except GNPS) locations. However, the GWAS panel was phenotyped for only three traits i.e. GWPS (gm), GY (gm), and PH (cm) at the Ludhiana location. Plant height (PH) was recorded at physiological maturity as the average of randomly selected three plants of each genotype by measuring from the soil surface to the spike tip excluding awns. Days to heading (DH) were recorded as the number of days from the planting when more than 50% of the plants in each plot showed the emergence of spikes. Physiological maturity was recorded when the majority of plants in the plot showed a complete loss of green colour from the flag leaf. The difference between the days to physiological maturity and the days to heading was used to compute the grain filling duration (GFD). Grain number per spike (GNPS) was calculated as the average of grain number in the main stem spikes of ten randomly selected plants from each genotype. Similarly, grains of all the randomly selected 10 spikes of each entry were weighed separately and the average of 10 spikes was recoded as grain weight per spike (GWS). Grain yield (GY) in grams for each genotype was recorded after harvesting the whole plot. Phenotypic data were analyzed using the R package ‘augmentedRCBD’ (Aravind et al., 2021).

### Genotyping and quality control (QC)

Cetyl Trimethyl Ammonium Bromide (CTAB) method (Murray and Thompson, 1980) was used to extract the genomic DNA from the leaves of 21 days-old seedlings. The GWAS panel was genotyped using Axiom Wheat Breeder’s Genotyping Array (Affymetrix, Santa Clara, CA, United States) having 35,143 genome-wide SNPs. The monomorphic, markers with minor allele frequency (MAF) of <5%, missing data of >20%, and heterozygote frequency >25% were removed from the analysis. The remaining set of 14,790 high-quality SNPs was used in GWAS analysis (Supplementary Table S2).

### Population statistics and GWAS

The pair-wise LD values (*r* ^2^) between the SNPs located in each chromosome were calculated with Trait Analysis by aSSociation Evolution and Linkage (TASSEL) version 5.0 (Bradbury et al., 2007). The LD block size in the whole genome and three subgenomes was estimated by keeping the *r* ^*2*^ threshold at half LD decay. The principal component analysis (PCA) and Kinship relationship were done through GAPIT (Lipka et al., 2012) to understand the structure of the population used in the GWAS model.

The phenotypic values of GWPS, GNPS, GY, DH, GFD, and PH of 280 diverse genotypes along with corresponding genotyping data were used in GWAS analysis. Significant MTAs were identified using the BLINK (Bayesian-information and Linkage-disequilibrium Iteratively Nested Keyway) model (Huang et al., 2019) implemented in Genome Association and Prediction Integrated Tool (GAPIT) version 3.0 (Wang and Zhang, 2021) in the R software package. Determining the correct *p*-value threshold for statistical significance is critical to differentiate the true positives from false positives. To determine the statistical significance threshold in GWAS, Bonferroni correction has been employed. To estimate Bonferroni correction, α was set to 0.05 which is divided by the total number of SNPs. The Bonferroni-corrected SNPs were considered for significant association and *R* ^2^ was used to describe the percentage variation explained (PVE) by significant MTAs.

### *In silico* analysis

The sequence information of the significant SNPs was used to search for putative candidate genes with the Basic Local Alignment Search Tool (BLAST) using default parameters in the Grain Gene database (https://wheat.pw.usda.gov/GG3/) of the bread wheat genome (Wheat Chinese Spring IWGSC RefSeq v2.1 genome assembly (2021)). The genes found in the overlapping region and within the region of 0.1 Mb intervals flanking either side of the associated marker were considered putative candidate genes and their molecular functions were determined. In addition, their expression patterns were investigated using the Wheat Expression database (http://www.wheat-expression.com/) and potential links to phenotypes were determined using the Knetminer tool integrated with the Wheat Expression database. The role of the identified putative candidate genes in the regulation of GWPS, GNPS, GY, DH, GFD, and PH was also determined by the previous reports.

## Results

The environment-wise heritability and variance components of the GWAS panel are presented in Table 1. All the studied traits recorded a wider distribution across the environments i.e. DH, GFD, GNPS, GWPS, PH, and GY ranging from 50.4 to 116.4 days, 19.6–55.3 days, 11.6–80.1 number, 0.2–4.6 gm, 57.6–134.8 cm, and 133.8–752.3 gm, respectively. The percent CV of all the studied environments is less than 10.0%, except GFD in E3 (11.1%) and GWPS in E2 (10.8%). The trend of heritability is more environment-specific than trait *per se*, as none of the environments recorded either only low or high performance for the studied traits. There is much variation in the trait’s heritability, which ranged from 50.5% to 97.2%.The genotypic variance ( $\sigma_{G}^{2}$ ) and environmental variance ( $\sigma_{E}^{2}$ ) are presented in Table 1.

**TABLE 1:** Descriptive statistics, variance, and heritability estimates of grain yield and component traits in GWAS panel evaluated at Dharwad, IARI Delhi, IARI Jharkhand, Karnal, and Ludhiana during 2020–2021.

Trait	Env.	Mean ± SD	Range	CV (%)	LSD	*h* ^*2*^_BS_	GV	EV
DH	E1	59.2 ± 3.8	50.4–72.2	2.4	4.1	85.2	11.9	2.1
	E2	95.8 ± 5.6	81.6–116.4	1.5	4.0	93.5	29.2	2.0
	E3	85.7 ± 4.8	69.8–98.8	1.0	2.3	97.2	23.1	0.7
	E4	94.7 ± 5.2	81.6–116.6	1.9	5.0	88.2	23.7	3.2
GFD	E1	45.0 ± 0.1	31.3–49.1	5.5	6.9	65.1	15.3	6.0
	E2	44.9 ± 0.1	26.9–51.9	4.9	6.2	60.5	13.5	4.8
	E3	31.6 ± 0.3	19.6–46.3	11.1	9.8	50.5	12.4	12.1
	E4	42.7 ± 0.3	28.3–55.3	5.6	6.7	70.9	13.7	5.6
GNPS	E1	52.3 ± 8.6	24.2–80.1	5.3	6.6	96.7	161.9	5.5
	E3	43.9 ± 12.9	11.6–79.4	4.1	6.0	93.9	70.6	4.6
GWPS	E1	2.1 ± 0.6	0.4–3.5	8.7	0.5	91.6	0.4	0.03
	E2	1.9 ± 0.6	0.2–3.9	10.8	0.6	88.3	0.3	0.04
	E3	2.4 ± 0.3	1.2–3.3	8.7	0.6	61.8	0.1	0.04
	E4	2.9 ± 0.5	1.5–4.6	8.9	0.8	73.4	0.2	0.07
	E5	2.1 ± 0.8	0.4–3.71	8.3	0.5	95.4	0.6	0.03
PH	E1	72.9 ± 6.9	57.6–94.1	2.5	5.2	92.9	44.9	3.4
	E2	102.2 ± 7.4	82.0–134.8	2.2	6.5	90.6	50.4	5.2
	E3	96.2 ± 7.6	77.5–115.8	2.2	5.9	91.5	46.4	4.3
	E4	110.8 ± 6.9	94.8–134.5	2.2	6.7	88.2	42.2	5.7
	E5	103.9 ± 5.9	81.3–120.1	2.5	7.2	80.6	27.0	6.5
GY	E1	312.7 ± 100.7	143.3–611.8	6.12	54.1	96.4	9835.2	366.1
	E2	466.8 ± 88.9	232.6–689.3	4.16	55.1	95.1	7348.2	379.6
	E3	256.3 ± 39.3	133.8–372.8	4.67	34.0	89.2	1199.6	144.9
	E4	556.9 ± 90.5	297.8–752.3	6.85	108.1	79.7	5746.2	1461.0
	E5	532.9 ± 82.8	243.2–734.2	5.1	77.2	88.9	5932.8	744.6

DH: days to heading (days); GFD: grain filling duration (days); GNPS: grain number per spike (number); GWPS: grain weight per spike (gm); PH: plant height (cm); GY: grain yield (gm). E1: Dharwad; E2: IARI, Delhi; E3: IARI, Jharkhand; E4: Karnal; E5: Ludhiana; SD: standard deviation; CV: coefficient of variation; *h* ^*2*^BS: broad sense heritability; GV: genotypic variation; EV: environmental variation.

**FIGURE 1:**
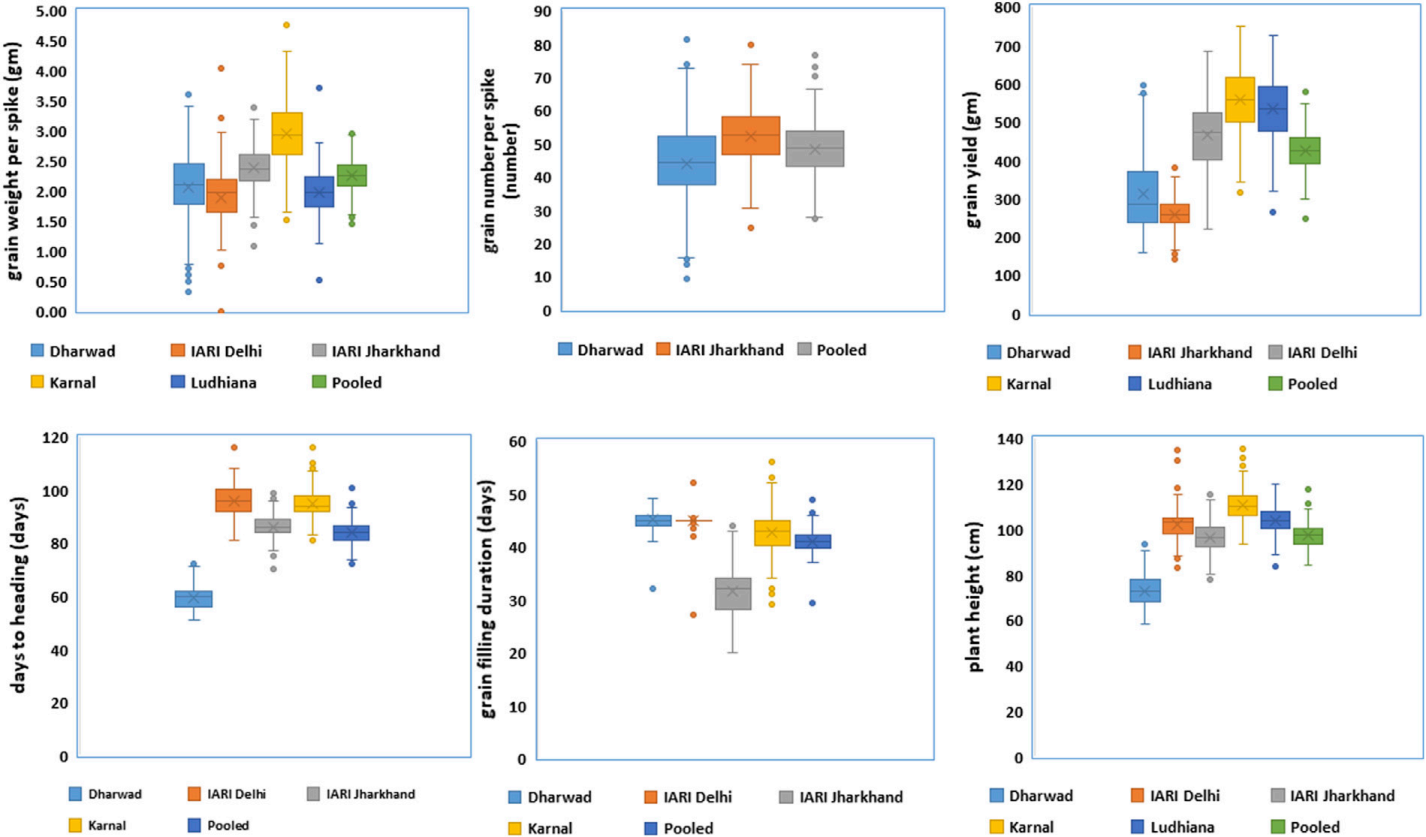
Boxplots of grain yield and component traits in GWAS panel evaluated at Dharwad, IARI Delhi, IARI Jharkhand, Karnal, and Ludhiana during 2020–2021.

**FIGURE 2:**
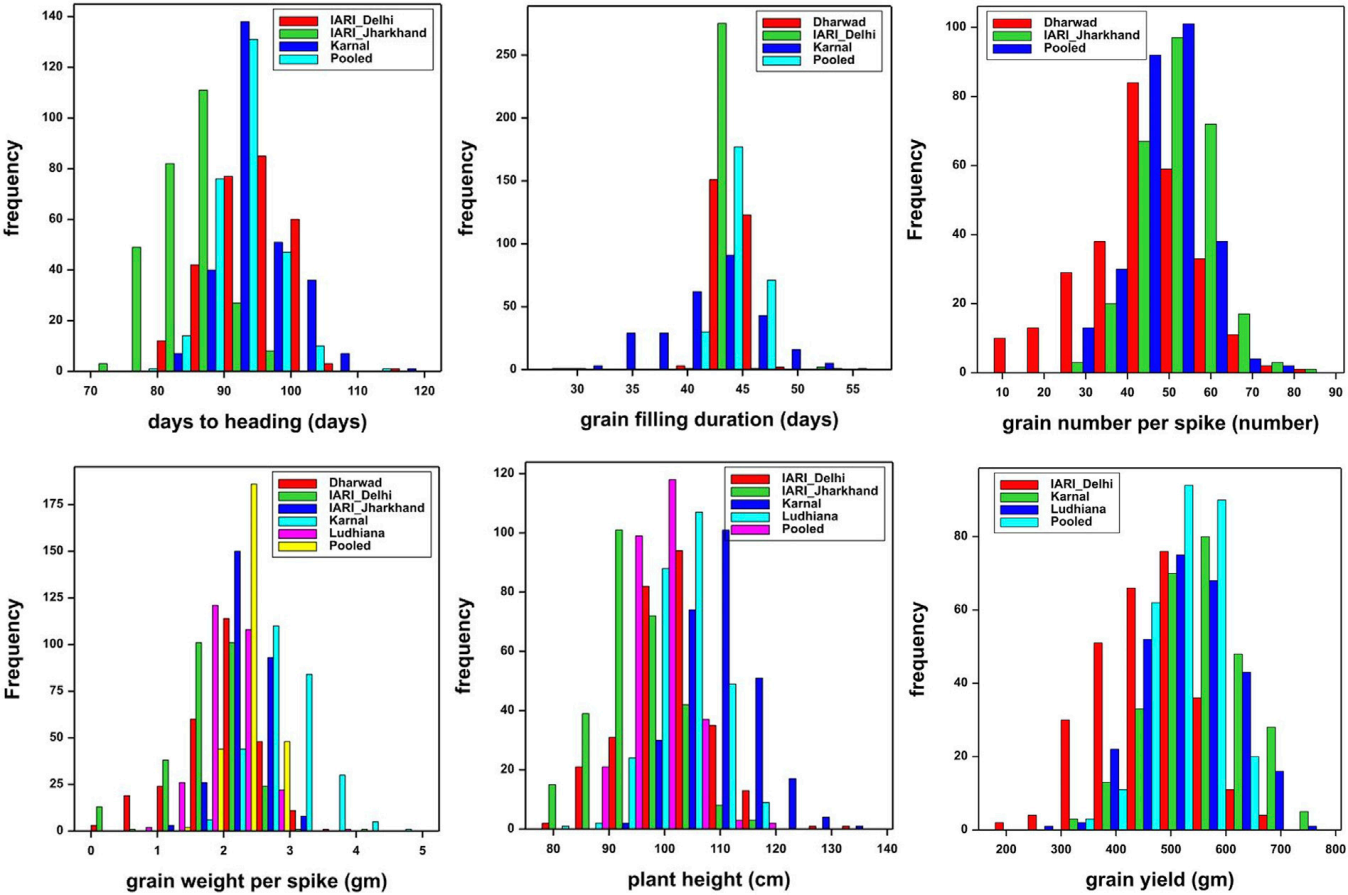
Frequency distribution of grain yield and component traits in GWAS panel evaluated at Dharwad, IARI Delhi, IARI Jharkhand, Karnal, and Ludhiana during 2020–2021.

The trait and environment-wise mean values are illustrated graphically through boxplots and presented in Figure 1. The location means of DH were recorded as similar and highest for E2 and E4 followed by E3, and E1, whereas, E1 and E2 were recorded as similar and highest followed by E4 and E3 for GFD. The E4 recorded the highest mean for GWPS followed by E3, E1, E5, and E2. The expression of PH is also similar to DH, as the highest and lowest are recorded by E4 and E1 respectively. The highest yields were recorded by E4 and E5, which is higher than the grand pooled mean, followed by E2, E1, and E3. The general yield trend of E4 and E5 are similar and higher than the pooled mean, the trend was exactly the opposite in E1 and E3 as both of them are similar, which are lower than the grand pooled mean. Whereas, the grain yield of E2 hovers around the pooled mean (Figure 1). The frequency distribution of grain yield and component traits in the GWAS panel evaluated at E1–E5 during 2020–2021 is presented in Figure 2. The genotypes in the GWAS panel showed continuous frequency distributions for all the studied traits. Pearson’s correlation coefficient (*r* ^*2*^) of DH, GFD, GNPS, GWPS, PH, and GY was determined and presented in Table 2. The grain-related traits i.e. GNPS and GWPS were a significant positive association with GY in all the environments and pooled mean except E2, where the association was neutral. A similar trend of significant positive association was observed between GY and PH in all the environments and pooled mean, except E4 and E5, where the association was neutral. However, the correlation between DH and GFD is consistent and significant negative in all the environments and pooled mean.

**TABLE 2:** Estimates of phenotypic correlation coefficients for grain yield and component traits in GWAS panel evaluated at Dharwad, IARI Delhi, IARI Jharkhand, Karnal, and Ludhiana during 2020–2021.

Environments	Traits	GWPS	GNPS	GY	DH	GFD	PH
Dharwad (E1)	GWPS	1.00	0.89**	0.45**	−0.25**	0.00	0.23**
	GNPS		1.00	0.30**	−0.11	0.01	0.13*
	GY			1.00	−0.11	−0.09	0.35**
	DH				1.00	−0.13**	0.00
	GFD					1.00	0.042
	PH						1.00
IARI-Delhi (E2)	GWPS	1.00	—	−0.04	0.050	−0.02	−0.02
	GY			1.00	0.04	0.00	0.13**
	DH				1.00	−0.22**	0.18**
	GFD					1.00	−0.09
	PH						1.00
IARI Jharkhand (E3)	GWPS	1.00	0.55**	0.27**	0.03	0.01	0.25**
	GNPS		1.00	0.12*	0.25**	−0.13*	0.18**
	GY			1.00	−0.01	−0.1	0.15*
	DH				1.00	−0.39**	0.10
	GFD					1.00	−0.29**
	PH						1.00
Karnal (E4)	GWPS	1.00	—	0.21**	−0.04	−0.00	0.25**
	GY			1.00	0.04	−0.08	0.06
	DH				1.00	−0.95**	0.21**
	GFD					1.00	−0.21**
	PH						1.00
Ludhiana (E5)	GWPS	1.00	—	0.89**	—	—	0.12
	GY			1.00	—	—	0.09
	PH						1.00
Across Environment	GWPS	1.00	0.61**	0.44**	−0.09	−0.07	−0.21**
	GNPS		1.00	0.31**	0.09	−0.19**	0.09
	GY			1.00	0.04	−0.03	0.18**
	DH				1.00	−0.71**	0.19**
	GFD					1.00	−0.25**
	PH						1.00

GWPS, grain weight per spike (gm); GNPS, grain number per spike (number); GY, grain yield (gm); DH, days to heading (days); GFD, grain filling duration (days); PH, plant height (cm).

### Genome-wide SNP markers distribution

The 35K SNP array was processed to obtain high-quality SNPs, as a result, a set of 14,790 cured genome-wide SNPs was selected. These high-quality set of SNPs along with phenotypic data were further used for GWAS analysis. The highest number of SNPs were mapped on the B subgenome (5649) followed by the D subgenome (4590), and the A subgenome (4551). Chromosome-wise highest SNPs were mapped on 1B (1077), followed by 2B (992), 1D (986), 2D (951), 5B (863), 6B (766), 7B (760), 2A (756), 1A (751), 7A (750), 3B (726), 5A (699), 5D (657), 3D (648), 7D (625), 3A (587), 6A (515), 4A (493), 4B (465), 6D (459), and 4D (264).

### Population structure and linkage disequilibrium

**FIGURE 3:**
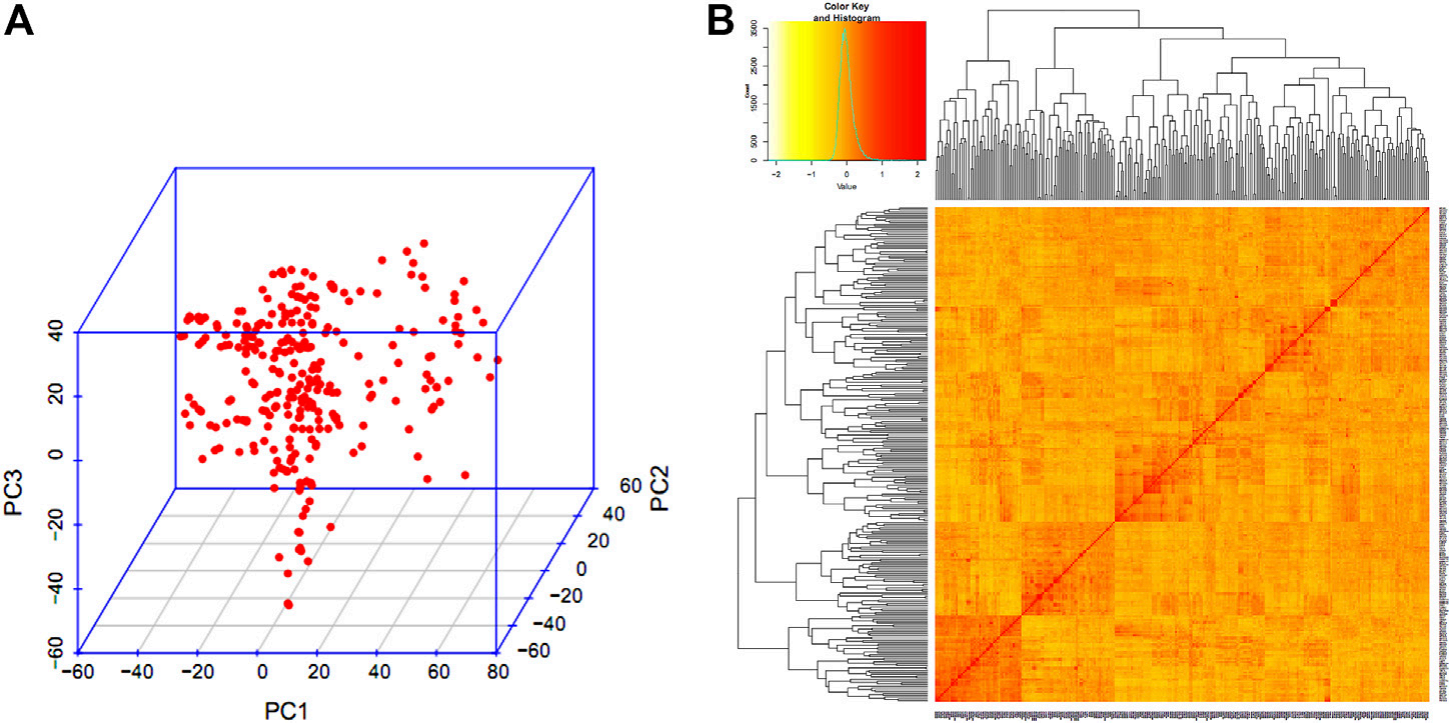
Population structure of GWAS panel. **(A)** Three-dimensional plot of the first three principal components. **(B)** Heat map of pair-wise kinship matrix.

The PCA analysis (Figure 3) indicated that there were no clear distinct sub-populations in the GWAS panel. The LD was estimated by calculating the squared correlation coefficient (r^2^) for all the SNPs. The LD decay for the whole genome was 4.9 cM and it was found that the decay was rapid in the A genome (3.6 cM) followed by the D genome (5.2 cM) and B genome (5.7 cM).

### Genome-wide association studies

**FIGURE 4:**
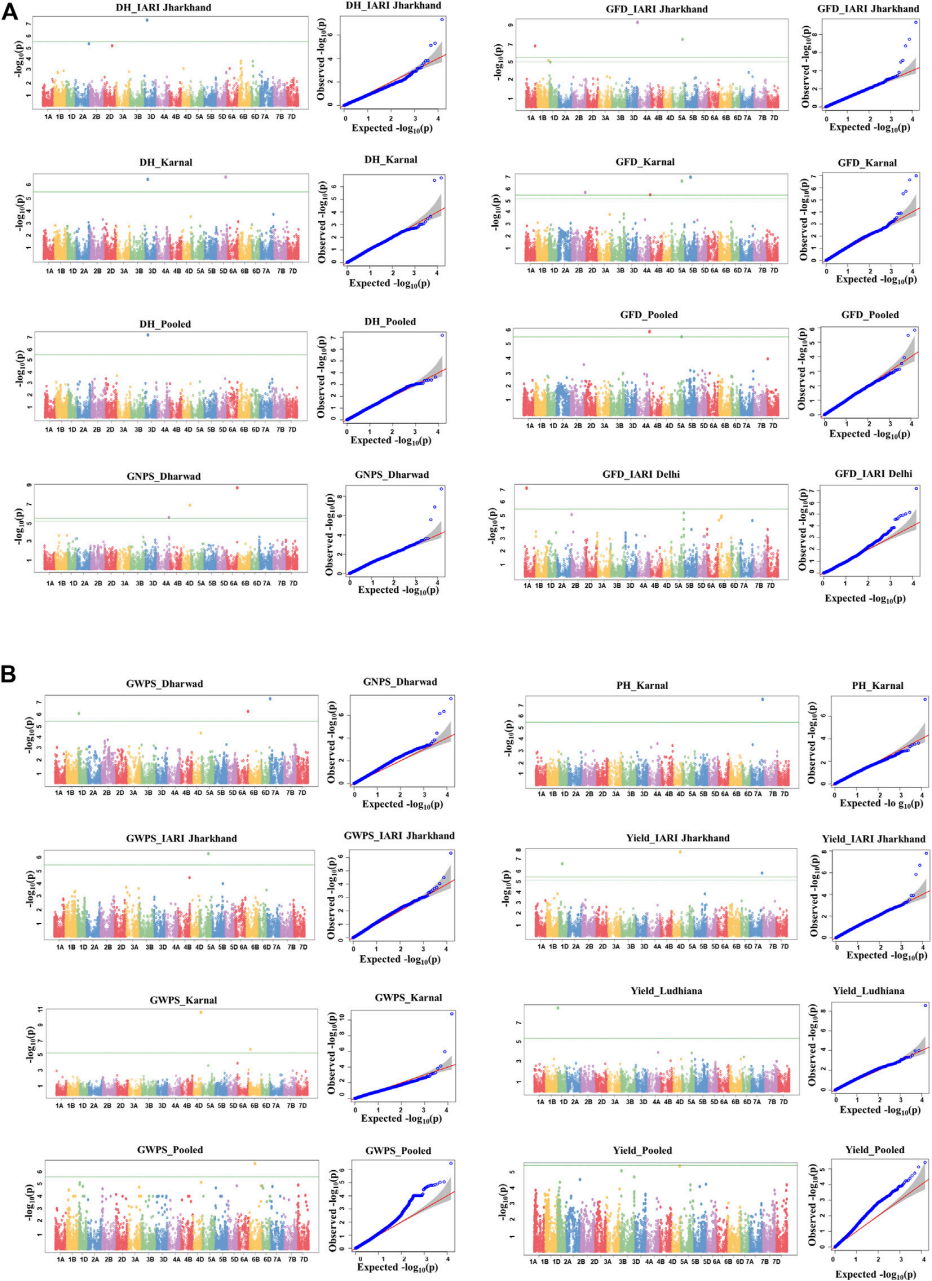
**(A)** Manhattan and respective-QQ plots for days to heading, grain number per spike, and grain filling duration in GWAS panel phenotyped at Dharwad, IARI Delhi, IARI Jharkhand, Karnal, and Ludhiana during 2020–2021. **(B)**Manhattan and respective-QQ plots for grain weight per spike, plant height, and yield in GWAS panel phenotyped at Dharwad, IARI Delhi, IARI Jharkhand, Karnal, and Ludhiana during 2020–2021.

A total of 27 Bonferroni-corrected MTAs were identified for DH, GFD, GNPS, GWPS, PH, and GY. The details of the identified MTAs are presented in Table 3 and illustrated in Manhattan plots in Figures 4A,B. The Q-Q plots depicting the observed associations of SNPs of DH, GFD, GNPS, GWPS, PH, and GY compared to the expected associations after accounting for population structure are presented in Figures 4A,B.

**TABLE 3:** Marker trait associations for grain yield and component traits in GWAS panel evaluated at Dharwad, IARI Delhi, IARI Jharkhand, Karnal, and Ludhiana during 2020–2021.

Trait	Environment	SNPs	Chr.	Position	p.value	PVE (%)
DH	E3	*AX-95024590*	3D	152,556,482	5.00E-08	21.1
	E4	*AX-94724456*	5D	484,426,977	2.04E-07	14.6
		*AX-95024590*	3D	152,556,482	3.17E-07	14.4
	Pooled	*AX-95024590*	3D	152,556,482	6.60E-08	17.3
GFD	E2	*AX-95107750*	1A	112,941,690	6.51E-08	9.1
	E3	*AX-94598412*	4A	5,068,136	4.67E-10	17.7
		*AX-95181791*	5A	584,672,964	3.44E-08	18.1
		*AX-94691261*	1A	505,524,324	1.82E-07	15.9
	E4	*AX-94794189*	5B	356,188,192	1.05E-07	18.2
		*AX-95210025*	5A	585,412,855	2.25E-07	19.0
		*AX-94702510*	2B	776,211,899	1.99E-06	19.9
		*AX-94425015*	4B	2,036,666	3.13E-06	20.4
	Pooled	*AX-94425015*	4B	2,036,666	1.47E-06	16.7
		*AX-95210025*	5A	585,412,855	3.37E-06	15.4
GNPS	E1	*AX-94539354*	6A	599,237,214	1.70E-09	16.3
		*AX-94978133*	4D	465,771,817	1.26E-07	11.1
		*AX-94658573*	4A	715,512,644	2.65E-06	10.1
	Pooled	*AX-94539354*	6A	599,237,214	2.26E-12	15.6
GWPS	E1	*AX-94602474*	7A	15,695,625	3.51E-08	11.4
		*AX-94539354*	6A	599,237,214	4.67E-07	17.1
		*AX-94883693*	1D	40,579,284	7.14E-07	14.0
	E3	*AX-94469473*	5A	521,025,733	4.76E-07	11.4
	E4	*AX-94978133*	4D	465,771,817	1.62E-11	12.5
		*AX-95105308*	6B	113,453,322	1.09E-06	6.6
	Pooled	*AX-94387482*	6B	337,941,809	3.39E-07	11.0
PH	E4	*AX-94452759*	2D	8,890,070	5.04E-08	5.4
		*AX-94498579*	3B	82,448,998	1.51E-07	5.0
		*AX-94796636*	5D	294,585,572	2.23E-06	7.2
GY	E3	*AX-94978133*	4D	465,771,817	1.66E-08	9.3
		*AX-94483483*	1D	206,101,173	2.09E-07	8.1
		*AX-94709904*	7A	728,244,062	1.49E-06	10.8
	E5	*AX-94473624*	1D	19,313,725	2.74E-09	12.5
	Pooled	*AX-94978133*	4D	465,771,817	3.87E-06	15.4

DH: days to heading (days); GFD: grain filling duration (days); GNPS: grain number per spike (number); GWPS: grain weight per spike (gm); PH: plant height (cm); GY: grain yield (gm); E1: Dharwad; E2: IARI, Delhi; E3: IARI, Jharkhand; E4: Karnal; E5: Ludhiana; SNPs: single nucleotide polymorphisms; PVE%: percent phenotypic variation explained. Gene and marker names were italicized.

### MTAs for grain-related traits

A total of seven significant MTAs were identified for GWPS in E1, E3, and E4 along with pooled mean on chromosomes 1D, 4D, 5A, 6A, 6B, and 7A, explaining the phenotypic variation ranging from 6.6% to 17.1%. The major MTA (*AX-94539354*) on 6A chromosome located at 599.2 Mb explained the highest phenotypic variation of 17.1%. Similarly, two more MTAs (*AX-94602474* and *AX-94883693*) were identified in E1, which were mapped at 15.6 Mb and 40.5 Mb and explained 11.4% and 14.0% of PVE, which were located on 7A and 1D, respectively. Two MTAs i.e. *AX-94978133* and *AX-95105308* were respectively mapped at 465.7 and 113.4 Mb on 4D and 6B chromosomes in the E4 environment. One MTA each on chromosome 6B (*AX-94387482*) and 5A (*AX-94469473*) were mapped at 337.9 and 521.0 Mb, respectively with the phenotypic variation of 11.0% and 11.4% at pooled mean and E3 environment. Genome-wise, a total of three significant MTAs were identified in the A genome while B and D genomes had two each.

A total of three significant MTAs were identified for GNPS in E1 and pooled mean on chromosomes 4A, 4D, and 6A. One MTA (*AX-94539354*) was mapped at 599.2 Mb on 6A in both E1 and pooled mean and explained 16.3% and 15.6% PVE, respectively. The remaining two MTAs (*AX-94658573* and *AX-94978133*) were identified in E1 and mapped at 715.5 and 465.7 Mb on 4A and 4D, explaining more than 10.0% PVE. Genome-wise, two significant MTAs were identified in the A subgenome and one in the D subgenome, however, there is no representation of the B subgenome.

For GY, four significant MTAs (*AX-94473624, AX-94483483, AX-94709904,* and *AX-94978133*) were identified on 1D, 4D, and 7A chromosomes in E3 and E5 along with pooled mean. Three MTAs i.e. *AX-94483483, AX-94709904,* and *AX-94978133* were identified in E3 and mapped at 206.1 Mb, 728.2 Mb, and 465.7 Mb with percent PVE of 8.1, 10.8, and 9.3, respectively. The remaining MTA (*AX-94473624*) was identified in E5 and mapped at 19.3 Mb with a percent PVE of 12.5. Also, one consistent MTA (*AX-94978133*) was identified in both E3 and pooled mean. Genome-wise, three significant MTAs were identified in the D subgenome and one in the A subgenome, however, there is no representation of the B subgenome.

### MTAs for agro-morphological traits

A total of two MTAs (*AX-94724456* and *AX-95024590*) were identified for DH in E4 and E3 along with pooled mean. One MTA i.e. *AX-95024590* was identified in two environments (E3 and E4) along with pooled mean. The other MTA (*AX-94724456*) was identified in E4 and mapped at 484.4 Mb on the 5D chromosome, which explained a PVE of 14.6%. All the MTAs explained more than 14.0% PVE and were located on the D subgenome only. For PH, all three MTAs (*AX-94452759, AX-94498579,* and *AX-94796636*) were identified in E4 with low percent PVE, which ranged from 5.0 to 7.2. Genome-wise, two significant MTAs were identified in the D subgenome and one in the B subgenome, however, there is no representation of the A subgenome.

The highest number of MTAs (8) were identified for GFD in E2, E3, and E4 along with pooled mean on 1A, 2B, 4A, 4B, 5A, and 5B chromosomes. A maximum of four MTAs i.e. *AX-94425015, AX-94702510, AX-94794189,* and *AX-95210025* were identified in E4 and mapped at 2.0 Mb, 776.2 Mb, 356.1 Mb, and 585.4 Mb on 4B, 2B, 5B, 5A chromosomes, respectively with PVE ranging from 18.2% to 20.4%. A total of three MTAs i.e. *AX-94598412, AX-94691261*, and *AX-95181791* were identified in E3 and mapped at 5.0 Mb, 505.5, and 584.6 Mb on 4A, 1A, and 5A chromosomes, respectively with PVE ranging from 15.9% to 18.1%. Two MTAs (*AX-94425015* and *AX-95210025*), which was identified for pooled mean, which was mapped at 2.0 and 585.4 Mb on 4B and 5A chromosomes, respectively with PVE of 16.7% and 15.4%. E2 environment is represented with one MTA (*AX-95107750*) on the 1A chromosome and mapped at 112.9 Mb with a PVE of 9.1%. Genome-wise, five significant MTAs were identified in the D subgenome and three in the B subgenome, however, there is no representation of the D subgenome.

### Stable and co-localized MTAs

A total of five consistent MTAs were identified on 3D, 4B, 5A, 6A, and 4D chromosomes for DH, GFD, GNPS, and GY. One MTA i.e. *AX-95024590* was identified in two environments (E3 and E4) along with pooled mean for DH, which was mapped at 152.5 Mb with PVE ranging from 14.4% to 21.1%. Similarly, two MTAs i.e. *AX-94425015* and *AX-95210025* were mapped at 2.0 and 585.4 Mb with the PVE ranging from 15.4% to 20.4%, respectively, which were consistently identified both in E4 and pooled mean for GFD. One each consistent MTA was identified for GNPS (*AX-94539354*) and GY (*AX-94978133*) on 6A and 4D chromosomes, respectively, which were mapped at 599.3 and 465.7 Mb with PVE ranging from 9.3% to 16.3%. The GNPS MTA was identified in both E1 and pooled mean, whereas, GY MTA was identified in E3 and pooled mean. Furthermore, two co-localized MTAs were identified on 6A and 3D chromosomes. One co-localized MTA (*AX-94978133*) was identified on the 4D chromosome for all the three-grain related traits (GNPS, GWPS, and GY), which was mapped at 465.7 Mb with the PVE ranging from 9.3% to 15.4%. The other co-localized MTA (*AX-94539354*) was identified on the 6A chromosome for GNPS and GWPS, which was mapped at 599.2 Mb with the PVE ranging from15.6% to 17.1%.

### Putative candidate genes associated with MTAs

The significant SNPs associated with GWPS, GNPS, GY, DH, GFD, and PH were used to identify the putative candidate genes using the annotated wheat reference sequence (Wheat Chinese Spring IWGSC RefSeq v2.1 genome assembly (2021)) and are presented in Table 4. *AX-94724456* associated with DH found to encode *F-box-like domain superfamily* (TraesCS5D03G0939000). Similarly, SNPs i.e. *AX-95107750, AX-94598412, AX-94598412, AX-95181791, AX-95181791, AX-94691261* associated with GFD and encodes *Lateral organ boundaries, LOB* (TraesCS1A03G0271300), *Thioredoxin-like superfamily* (TraesCS4A03G0014500), *Glutathione S-transferase* (TraesCS4A03G0014500), *RNA-binding domain superfamily* (TraesCS5A03G0929100), *UDP-glycosyltransferase family* (TraesCS5A03G0929200), *Serine/threonine-protein kinase* (TraesCS1A03G0779800), respectively. Another SNP (*AX-94978133*) associated with GNPS found to encode *Expansin* (TraesCS4D03G0700700). Also, one SNP i.e. *AX-94883693* associated with GWPS found to encode *Patatin* (TraesCS1D03G0131800). Two SNPs (*AX-94452759* and *AX-94796636*) associated with PH found to encode *DUF1618 domain* (TraesCS2D03G0036000) and *Protein kinase domain* (TraesCS5D03G0455800). Similarly, *AX-94473624* associated with GY found to encode *Exocyst complex component Exo70* (TraesCS1D03G0081100).

**TABLE 4:** Putative candidate genes for grain yield and component traits.

Trait	SNP ID	Position	TransID	Putative candidate genes	Function
DH	*AX-94724456*	5D:487240908..487243412 (+strand)	TraesCS5D03G0939000	*F-box-like domain superfamily*	Heat Stress tolerance in wheat (Li et al., 2018). Regulate root growth and abiotic stress tolerance in rice (Yan et al., 2011)
GFD	*AX-95107750*	1A:115131649..115133537 (- strand)	TraesCS1A03G0271300	*Lateral organ boundaries, LOB*	Floral organs development in Arabidopsis (Shuai et al., 2002). Stress tolerance in potato (Liu et al., 2019a) and wheat (Wang et al., 2021)
	*AX-94598412*	4A:5076587..5077492 (- strand)	TraesCS4A03G0014500	*Thioredoxin-like superfamily*	Seed germination (Guo et al., 2013) and disease resistance in wheat (Shi et al., 2021)
	*AX-94598412*	4A:5076587..5077492 (- strand)	TraesCS4A03G0014500	*Glutathione S-transferase*	Growth and development, salt and drought stress tolerance in wheat (Wang et al., 2019)
	*AX-95181791*	5A:586571409..586575276 (+strand)	TraesCS5A03G0929100	*RNA-binding domain superfamily*	Extend grain filling duration and improve malt barley agronomic performance (Alptekin et al., 2021)
	*AX-95181791*	5A:586576183..586579881 (+strand)	TraesCS5A03G0929200	*UDP-glycosyltransferase family*	Regulation of grain size and abiotic stress tolerance in Rice (Dong et al., 2020)
	*AX-94691261*	1A:507053551..507056672 (+strand)	TraesCS1A03G0779800	*Serine/threonine-protein kinase, active site*	High thousand kernel weight and grains per spike in wheat (Ur Rehman et al., 2019)
GNPS	*AX-94978133*	4D:465931660..465932025 (- strand)	TraesCS4D03G0700700	*Expansin*	Capsule number in tobacco (Chen et al., 2016). Grain size in wheat (Lizana et al., 2010; Calderini et al., 2020)
GWPS	*AX-94883693*	1D:43497907..43499982 (+strand)	TraesCS1D03G0131800	*Patatin*	Seed size in Arabidopsis (Huang et al., 2001)
GY	AX-94473624	1D:20838814..20851719 (+strand)	TraesCS1D03G0081100	*Exocyst complex component Exo70*	Tissue-specific expression in wheat for biotic and abiotic stress (Zhao et al., 2018). Role in seed development in soybean (Wang et al., 2016). Pollen development in Arabidopsis (Markovic et al., 2020)
PH	AX-94452759	2D:9070785..9073543 (+strand)	TraesCS2D03G0036000	*DUF1618 domain*	Role in development and fitness in rice (Wang et al., 2014)
	AX-94796636	Chr5D:297449170..297458745 (+strand)	TraesCS5D03G0455800	*Protein kinase domain*	OstMAPKKK5 regulates plant height and yield in rice (Liu et al., 2019)

DH: days to heading (days); GFD: grain filling duration (days); GNPS: grain number per spike (number); GWPS: grain weight per spike (gm); PH: plant height (cm); GY: grain yield (gm). Gene and marker names were italicized.

## Discussion

Although the phenotype-based selection in conventional breeding has improved wheat yield for several decades, genotype-based strategies may further complement the varietal improvement programmes. Recent efforts to sequence the wheat genome could promote the rapid improvement of varieties through molecular breeding by using genetic resources. In wheat, many QTLs/MTAs have been identified for yield and component traits, however additional genetic studies are warranted using different genetic materials, as we have not reached a saturation point (Singh et al., 2021). Due to the genetic complexity of the wheat genome, there is always possibility to identify novel genomic regions with different genetic materials. Understanding the genetic basis of complex traits such as grain yield and component traits through GWAS with a diverse panel of genotypes can significantly improve QTL mapping resolution compared to bi-parental populations-based QTL mapping. Using the genome-wide SNPs and multi-environment data, several significant SNPs were identified in this study. Furthermore, stable and co-localized MTAs were also identified.

The significant effect of environment and genotype-environment interactions (GEI) was observed in the expression of all the studied traits. Among all traits, GFD was the most environment-sensitive trait, whereas, GNPS was relatively the most stable with minimum environmental influence. The greater magnitude of the environment and GEI have also been reported in previous studies for the expression of yield and component traits in wheat (Eltaher et al., 2021; Malik et al., 2021; Said et al., 2022). The GWAS panel has been tested in diverse production conditions, as the magnitude of GEI is a key factor in the identification of environment-specific QTL(s) as well as stable QTL(s). The highest and lowest heritability was recorded for GNPS and GFD, respectively, the trend for the percent contribution of environmental variation for the expression of GNPS and GFD was also exactly similar. Generally, the grain-related traits (GNPS and GWPS) and PH showed a significant positive association with GY in all the environments and pooled mean. However, the association between DH and GFD is consistent and significant negative in all the environments and pooled mean. The strong positive association of GY with GNPS, GWPS, and PH was further supported by the identification of two co-localized MTAs (*AX-94978133* and *AX-94539354*), associated with the same traits i.e. GNPS, GWPS, and GY*.* Significant correlations found in this study have also been reported in earlier studies (Juliana et al., 2018; Baye et al., 2020; Ullah et al., 2021). In crop improvement programmes, significant associations of yield and component traits in desired direction are always beneficial for simultaneous improvement of the associated traits. Furthermore, for negatively associated traits, it is advised to adopt breeding methods that could break the undesirable linkages, so that the traits can be independently improved.

PCA result of the filtered SNP data showed the allele frequencies of the genotypes were evenly distributed without any distinct sub-populations in the GWAS panel. The even distribution of allele frequencies in the GWAS panel was achieved by carefully selecting advanced breeding lines for different wheat growing zones in India, representing five agro-ecological zones, namely the Northern Hills Zone and North Western-Plains, North-Eastern Plains Zone, Central Zone, and Peninsular Zone. The various factors including size of the population, genetic drift, admixtures, selection, mutation, non-random mating, pollination behavior, and recombination frequency may affect the LD, therefore, LD may vary in different populations (Gupta et al., 2005; Vos et al., 2017). Self-pollinated crops like wheat usually have larger LD blocks and hence decay slowly (Yu et al., 2014), whereas, LD decays rapidly in outcross crop species such as maize (Dinesh et al., 2016). The presence of high LD across the genome would reduce the QTL mapping resolution and vice versa (Dadshani et al., 2021). Under such situations, a better QTL resolution may be achieved by using genome-wide SNPs. The LD decay was found to be high and comparable in the B and D subgenomes (∼5 cM) compared to the A subgenome, which had a shorter decay distance of around ∼3 cM. A similar pattern of LD decay was also observed in other GWAS studies in wheat (Sukumaran et al., 2015; Rahimi et al., 2019; Sheoran et al., 2019). Therefore, marker density and population size are two important determinants in GWAS studies and vary in self and cross-pollinated crops due to varied LD decay.

A total of 27 Bonferroni-corrected MTAs were identified for GWPS (7), GNPS (3), GY (4), DH (2), GFD (8), and PH (3). The highest number of MTAs were identified for A subgenome (11) followed by the D subgenome (10) and the B subgenome (6). A similar trend on MTAs identified in the A subgenome for yield and yield-contributing traits (Ain et al., 2015; Godoy et al., 2018; Alemu et al., 2021). A high level of stringency through Bonferroni-correction has been followed to consider MTA as significant, therefore, these MTAs could be valuable for their further validation in different genetic backgrounds to use them in MAS.

The identified MTAs (7) for GWPS on chromosomes 1D, 4D, 5A, 6A, 6B, and 7A in this study were novel as the earlier reported MTAs on the same chromosomes were identified at different positions. Although many grain-related traits like thousand kernel weight have been thoroughly studied, GWPS is comparatively less explored. Edae et al. (2014) identified an MTA on the 1D chromosome located at 88.5 cM and on the 7A chromosome located at 107.1 cM. A total of three significant MTAs were identified for another grain-related trait i.e. GNPS on 4A (599.2 Mb), 4D (715.5 Mb), and 6A (465.7 Mb) chromosomes. MTAs for GNPS in the different chromosomes were identified in different GWAS panels in previous experiments (Edae et al., 2014; Sun et al., 2017; Godoy et al., 2018; Jamil et al., 2019). However, Russell et al. (2020) identified an MTA in the same chromosome of 4D at 479.5 Mb, which was similar to that of *AX-94978133* located on the 4D chromosome and mapped at 465.7 Mb, which explained 11.1% of phenotypic variation. All the identified MTAs explained more than 10% PVE for both the traits (GNPS and GWPS) except *AX-95105308*, which explained only 6.6% PVE.

The fundamental breeding objective of any wheat breeding program is the higher gains for GY, a highly complex and environmentally-sensitive economic trait. In the present study, a total of four significant MTAs were identified on 1D, 4D, and 7A chromosomes for GY. MTAs in the same chromosomes were also identified in different GWAS panels in previous studies on 1D (Bajgain et al., 2019; Jamil et al., 2019), 7A (Jamil et al., 2019; Ward et al., 2019; Russell et al., 2020) at different chromosomal locations. However, one MTA (S7A_720744946) on 7A chromosome was mapped at 720.7 Mb position, which is similar to the MTA identified in the present study i.e. (*AX-94709904*) on the 7A chromosome, which was mapped at 728.2 Mb. Furthermore, MTAs for GY were also identified in different chromosomes in different GWAS studies with diverse genetic material (Edae et al., 2014; Ain et al., 2015; Godoy et al., 2018; Li et al., 2019; Rahimi et al., 2019; Alemu et al., 2021; Suliman et al., 2021).

GWAS of yield component traits including DH, PH, and GFD led to the detection of 13 genetic loci associated with these traits. Two MTAs were identified for DH on 3D (152.5 Mb) and 5D (484.4 Mb) chromosomes. Previous reports identified MTAs mostly on different chromosomes, for instance, 3B (Edae et al., 2014; Ain et al., 2015; Gahlaut et al., 2019; Russell et al., 2020), 2D (Jamil et al., 2019), 1A, 4A, 5A, and 6A (Godoy et al., 2018) chromosomes. Three MTAs for PH were identified on 2D (88.9 Mb), 3B (824.4 Mb), and 5D (294.5 Mb) chromosomes. Previous reports also identified MTAs on the same chromosomes 2D (Ward et al., 2019; Alemu et al., 2021), 3B (Edae et al., 2014; Gahlaut et al., 2019) and also on different chromosomes (Ain et al., 2015; Sun et al., 2017; Godoy et al., 2018; Li et al., 2019; Russell et al., 2020) for PH. Jamil et al. (2019) identified an MTA on the same chromosome 3B at 824.6 Mb, which was similar to that of *AX-94498579* located on the 3B chromosome and mapped at 824.4 Mb. The rate of grain filling and the length of grain filling period are two important determinants of final grain yield under different production conditions. In the present study, the maximum number of MTAs (8) was identified for GFD on 1A, 2B, 4A, 4B, 5A, and 5B chromosomes. Previous studies also identified on same chromosomes i.e. 1A (Edae et al., 2014; Jamil et al., 2019), 2B (Jamil et al., 2019), 5B (Edae et al., 2014; Jamil et al., 2019; Alemu et al., 2021) but different positions and different chromosomes (Godoy et al., 2018; Rahimi et al., 2019) for GFD.

A total of two co-localized MTA were identified, which are associated with multiple traits including GNPS, GWPS, GY, and DH. One co-localized MTA (*AX-94978133*) was identified on 4D associated with three traits (GNPS, GWPS, and GY). This MTA encodes *expansin* genes found to have a key role in wheat grain growth dynamics in wheat (Lizana et al., 2010; Calderini et al. (2020), increasing capsule number in tobacco (Chen et al., 2016). The other co-localized MTA (*AX-94539354*) identified on 6A was associated with two traits (GNPS and GWPS). Pleiotropic MTAs that are associated with multiple traits were also identified for grain yield and the biological yield on 1A, 4B, and 6B (Ain et al., 2015). Similarly, Co-localized QTLs associated with yield and component traits were detected (Alemu et al., 2021; Bennani et al., 2022). Such co-mapped SNPs will be much useful in marker-assisted selection for simultaneous improvement of correlated traits. Similarly, five consistent MTAs were also identified for grain yield and component traits in the present study. These co-located and stable MTAs will be suitable candidates for further validation and utilization in MAS-based varietal improvement programmes.

The various putative candidate genes underlying MTAs with high phenotypic variation for DH, GFD, GNPS, and GWPS were identified through BLAST search (Table 4). The MTAs identified in various chromosomes were located in gene coding regions related to transcription factors, a transmembrane protein, and kinase-like superfamilies. For example, AX-94978133 associated with GNPS encodes *expansin* (TraesCS4D03G0700700) genes found to have a role in wheat grain growth dynamics including grain size (Lizana et al., 2010). Calderini et al. (2020) demonstrated that the targeted over-expression of an α-expansin in early developing wheat seeds leads to a significant increase in grain size without a negative effect on grain number, resulting in a yield boost under field conditions. Similarly, constitutive expression of *TaEXPA2*, an α-expansin gene in tobacco improved seed production by increasing capsule number without having any effect on plant growth patterns (Chen et al., 2016).

One SNP i.e. *AX-95181791* associated with GFD encodes an important RNA-binding domain superfamily (TraesCS5A03G0929100) that extends grain filling duration in barley. Glycine-rich RNA-binding protein (*HvGR-RBP1*) and a NAC transcription factor (*HvNAM1*) extend grain filling duration and improve agronomic performance in malt barley (Alptekin et al., 2021). Similarly, *AX-94691261* was associated with GFD encodes *Serine/threonine-protein kinase* (TraesCS1A03G0779800). The role of wheat protein kinase gene i.e. *TaSnRK2.9-5A* was studied and found to be significantly associated with high thousand kernel weight, whereas, *Hap-5A-4* was associated with high grains per spike (Ur Rehman et al., 2019). Another SNP (*AX-95181791*) for GFD encoding *UDP-glycosyltransferase family* (TraesCS5A03G0929200) has also been identified. The role of *UDP-glucosyltransferase* studied by Dong et al. (2020) suggests that *UDP-glucosyltransferase* regulates grain size and abiotic stress tolerance in rice. One MTA (*AX-95107750*) on 1A associated with GFD which encodes *Lateral organ boundaries, LOB* (TraesCS1A03G0271300) has a role in floral organs development in Arabidopsis (Shuai et al., 2002). One MTA (AX-94883693) on a 1D chromosome associated with GWPS encodes *Patatin* (TraesCS1D03G0131800). The role of *Patatin* was studied by Huang et al. (2001) and found its role in seed size in Arabidopsis. One SNP (*AX-94473624*) on the 1D chromosome associated with grain yield encodes *Exocyst complex component Exo70* (TraesCS1D03G0081100) has been found to have a role in plant growth and development including tissue-specific expression in wheat for biotic and abiotic stress (Zhao et al., 2018), seed development in soybean (Wang et al., 2016), and pollen development in Arabidopsis (Markovic et al., 2020). Similarly, two SNPs (*AX-94452759* and *AX-94796636*) on 2D and 5D chromosomes associated with plant height encodes the *DUF1618 domain* (TraesCS2D03G0036000) and the *protein kinase domain* (TraesCS5D03G0455800) are involved in various plant developmental processes. *DUF1618 domain* has been found to have a role in the development and fitness of rice (Wang et al., 2019), and *protein kinase domain* regulates plant height and yield in rice (Liu Y. et al., 2019).

## Conclusion

The study with 280 diverse set of bread wheat GWAS panel has shown that DH, GFD, GNPS, GWPS, PH, and GY were quantitatively inherited traits. The strong positive correlation between GY and GNPS, GWPS, and PH suggested the possibility of improving these traits simultaneously. A total of 27 MTAs including 7 for GWPS, 3 for GNPS, 4 for GY, 2 for DH, 8 for GFD, and 3 for PH were identified through the GWAS approach. A total of five stable MTAs were identified in more than one environment and associated with the expression of DH, GFD, GNPS, and GY. Also, two novel pleiotropic genomic regions harboring co-localized QTLs governing two or more traits were also identified. The environment-specific and pooled-data MTAs identified in the present investigation represented novel genomic regions associated with trait expression. Several putative candidate genes encoding important molecular functions such as regulation of grain size, grain number, growth and development, grain filling duration, and abiotic stress tolerance were identified. Further validation and functional characterization of the candidate genes to elucidate the role of these genes in wheat is envisaged. The identified SNPs, particularly stable (*AX-95024590*, *AX-94425015, AX-95210025*, *AX-94539354, AX-94978133*) and pleiotropic SNPs (*AX-94978133* and *AX-94539354*) could be useful in marker-assisted selection programs to develop wheat varieties with increased grain yield.

## Data Availability

The datasets presented in this study can be found in online repositories. The names of the repository/repositories and accession number(s) can be found below: https://datadryad.org/stash/share/i2XTnmGIHO6F-LIlTjUhN1krZWActEJxWcZIbBSHAGo.

## Supplementary material

SUPPLEMENTARY TABLE 1 List of 280 genotypes used in the study along with pedigree and other details are given as Supplementary Table S1.

SUPPLEMENTARY TABLE 2 Genotyping and phenotyping information of the 280 genotypes used in the GWAS are given as Supplementary Table S2 and also deposited in DRYAD (an international open-access repository of research data). The international open-access data repository link is as follows: https://datadryad.org/stash/share/i2XTnmGIHO6F-LIlTjUhN1krZWActEJxWcZIbBSHAGo.
